# Global and Targeted Metabolomics for Revealing Metabolomic Alteration in Niemann-Pick Disease Type C Model Cells

**DOI:** 10.3390/metabo14100515

**Published:** 2024-09-24

**Authors:** Masahiro Watanabe, Masamitsu Maekawa, Keitaro Miyoshi, Toshihiro Sato, Yu Sato, Masaki Kumondai, Masayoshi Fukasawa, Nariyasu Mano

**Affiliations:** 1 Graduate School of Pharmaceutical Sciences, Tohoku University, 1-1 Seiryo-Machi, Aoba-Ku, Sendai 980-8574, Japanmano@hosp.tohoku.ac.jp (N.M.); 2 Department of Pharmaceutical Sciences, Tohoku University Hospital, 1-1 Seiryo-Machi, Aoba-Ku, Sendai 980-8574, Japan; toshihiro.sato@tohoku.ac.jp (T.S.); yu.sato.e7@tohoku.ac.jp (Y.S.); masaki.kumondai.d5@tohoku.ac.jp (M.K.); 3 Faculty of Pharmaceutical Sciences, Tohoku University, 1-1 Seiryo-Machi, Aoba-Ku, Sendai 980-8574, Japan; 4 Advanced Research Center for Innovations in Next-Generation Medicine, Tohoku University, 1-1 Seiryo-Machi, Aoba-Ku, Sendai 980-8574, Japan; 5 Department of Biochemistry and Cell Biology, National Institute of Infectious Diseases, 1-23-1 Toyama, Shinjuku-Ku, Tokyo 162-8640, Japan; fuka@niid.go.jp

**Keywords:** Niemann-Pick disease type C, metabolomics, amino acids, model cells, LC-MS/MS

## Abstract

Background: Niemann-Pick disease type C (NPC) is an inherited disorder characterized by a functional deficiency of cholesterol transport proteins. However, the molecular mechanisms and pathophysiology of the disease remain unknown. Methods: In this study, we identified several metabolite characteristics of NPC that may fluctuate in a cellular model of the disease, using both global and targeted metabolomic analyses by liquid chromatography/tandem mass spectrometry (LC-MS/MS). Three cell lines, HepG2 cells (wild-type[WT]) and two NPC model HepG2 cell lines in which *NPC1* was genetically ablated (knockout [KO]1 and KO2), were used for metabolomic analysis. Data were subjected to enrichment analysis using the Kyoto Encyclopedia of Genes and Genomes (KEGG) pathways. Results: The enrichment analysis of global metabolomics revealed that 8 pathways in KO1 and 16 pathways in KO2 cells were notably altered. In targeted metabolomics for 15 metabolites, 4 metabolites in KO1 and 10 metabolites in KO2 exhibited statistically significant quantitative changes in KO1 or KO2 relative to WT. Most of the altered metabolites were related to creatinine synthesis and cysteine metabolism pathways. Conclusions: In the future, our objective will be to elucidate the relationship between these metabolic alterations and pathophysiology.

## 1. Introduction

Niemann-Pick disease type C (NPC) is an autosomal recessive genetic disorder caused by a functional defect in a protein involved in cholesterol transport within lysosomes [1,2,3]. The pathophysiology of this disease is hypothesized to be caused by genetic mutations in the transporter membrane protein NPC1 [4] or the soluble binding protein NPC2 [5], which are involved in the transport and excretion of cholesterol. Both NPC1 and NPC2 recognize and bind to cholesterol. Therefore, mutations in *NPC1* or *NPC2* may cause various symptoms. Mutations in the *NPC1* gene are identified in approximately 95% of NPC cases, and mutations in the *NPC2* gene are identified in approximately 5% of NPC cases [6]. These symptoms manifest from infancy to adulthood. The clinical symptoms of this condition span a wide range, from visceral symptoms such as hepatosplenomegaly, jaundice, and pulmonary infiltration, which manifest during perinatal and infant periods, to chronic neurodegenerative symptoms that emerge in adulthood [7,8,9]. Neurological symptoms do not manifest in the neonatal period; however, they may emerge as the disease progresses [10,11]. Consequently, the clinical spectrum of NPC is extensive and heterogeneous, with a considerable proportion of patients presenting with neurological symptoms associated with progressive deterioration of the central nervous system following disease onset and succumbing to the illness within a few years of becoming severely ill [12,13,14]. Additionally, a plethora of neurological symptoms may manifest, including cerebellar ataxia, vertical gaze palsy, dysarthria, dysphagia, dementia, dystonia, and myoclonus. Currently, several treatment strategies are being investigated as potential treatments for NPC with the aim of slowing disease progression. Miglustat is an approved, disease-modifying drug [15,16,17]. Miglustat inhibits glucosylceramide synthase, thereby reducing glycosphingolipid levels and inhibiting neuronal loss. However, the efficacy of this treatment is limited to patients with advanced neurological symptoms. Therefore, early diagnosis and prompt treatment are crucial. Several biomarkers of NPC have been identified [18,19,20]. Alternative treatments, such as *N*-acetyl-L-leucine [21] and 2-hydroxypropyl-β-cyclodextrin [22], are also being developed, although they do not cure NPC.

Although NPC is a genetic disease, its pathological and molecular mechanisms remain poorly understood. There is evidence of damage to internal organs, accompanied by neurological symptoms and the enlargement of the liver and spleen. However, the molecular pathological mechanisms that lead to the development of these conditions remain largely unknown [23]. The impairment of cholesterol transport and accumulation of cholesterol-related lipids in the late endosome/lysosome compartment have been proposed as mechanisms that contribute to the development of this disease [8]. Although the specific genetic mutations that cause this condition are diverse [24,25,26,27], the precise correlation between these mutations and clinical phenotypes remains unclear [11]. Therefore, it is essential to comprehensively analyze these molecular alterations. This study focused on alterations in the metabolome, a collective term for low-molecular-weight metabolites with a molecular weight of less than 1,000, produced as metabolic products during vital activities [28,29,30,31]. The metabolome is influenced by upstream biomolecules, including DNA, RNA, and proteins [32,33]. Moreover, the metabolome can be used for the diagnosis, prevention, and evaluation of disease treatment efficacy [34,35,36,37]. The discovery of metabolic abnormalities has led to the development of new diagnostic biomarkers [18,19,20]. Some metabolites have been used as NPC biomarkers. Oxysterols produced from cholesterol were among the first biomarkers used [38,39]. Oxysterols can also produce abnormal bile acids [40,41,42]. Bile acids are also found in the urine [36,41,43]. Sphingolipids and their related metabolites have also been used as biomarkers [37,44,45,46,47,48]. *N*-Palmitoyl-*O*-phosphocholine-serine (PPCS), which was previously regarded as an analog of lysosphingomyelin, is an excellent and highly accurate biomarker [19,37,46,48,49,50,51]. Sphingolipids and PPCS are produced from L-serine and fatty acids [52,53]. However, the specific altered pathways in NPC metabolism remain unclear and there is a possibility of underlying metabolic irregularities.

**Figure 1:**
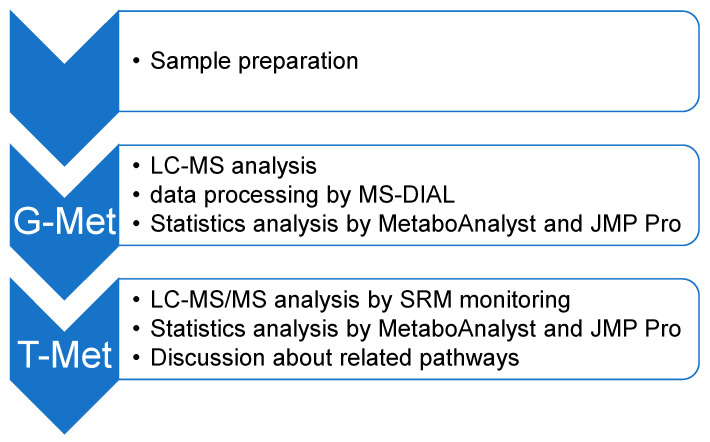
The metabolic analytical workflow of this study. First, we prepared the cell samples. Next, global metabolomics analysis is performed to identify alterations in the NPC model cells. Finally, the metabolites are quantified using targeted metabolomics, and the disease-related pathways are explored.

Therefore, in this study, we conducted a metabolome analysis to elucidate the extensive metabolic alterations observed in NPC (Figure 1). As an NPC model, we used a human hepatic cell line (HepG2)-based NPC1 deficient model. As described previously, we used two NPC cell lines at the different mutation sites in the *NPC1* sequence [54]. We showed both different and common alterations compared to the wild-type (WT) cell line. Among the common alternative pathways, ferroptosis and mytophagy are new NPC pathologies [54].

In this study, two distinct methodologies were employed to determine precise alterations. Global metabolomics (G-Met) is a comprehensive approach that allows for the analysis of several unidentified components without imposing limitations on the molecules to be measured [55,56,57]. Moreover, it is an effective instrument for analyzing metabolic irregularities in a spectrum of ailments and identifying biomarkers [58,59]. Liquid chromatography/mass spectrometry (LC/MS) was used to analyze G-Met levels. However, the G-Met approach is not quantitative [60]. Although the quantitative analysis provides accurate results, it has certain limitations. One such limitation is the potential for variability within and between analysis batches. Targeted metabolomics (T-Met) was used to enhance the efficacy of this approach. This study employed a comprehensive analysis of the metabolic changes in cultured NPC model cells using G-Met [54,61]. Finally, alterations in some metabolites observed using G-Met were quantified using T-Met and fluctuating metabolic pathways were investigated using the Kyoto Encyclopedia of Genes and Genomes (KEGG) [62]. KEGG is a comprehensive database that integrates a wide range of biological data to understand functions or biological systems using molecular-level information. Therefore, we analyzed cells using both G-Met and T-Met and applied the data to the KEGG database to analyze alterations in the metabolome in NPC pathology (Figure 1).

## 2. Materials and Methods

### 2.1. Chemicals and Reagents

L-Cysteine, L-methionine, L-proline, creatinine, L-glutamine, L-tryptophan, L-arginine monohydrochloride, L-tyrosine, L-cysteine were purchased from Nacalai Tesque (Kyoto, Japan). L-Carnitine, L-glutamic acid, hydrochloric acid, glutathione, arginine-[^13^C_6_,^15^N_4_] hydrochloride, formic acid, and 28% aqueous ammonia were purchased from FUJIFILM Wako Pure Chemicals (Osaka, Japan). L-serine, L-ornithine monohydrochloride, and glycocyamine were purchased from Tokyo Chemical Co. (Tokyo, Japan). Creatine-^2^H_3_ was purchased from Cambridge Isotope Laboratories (Tewksbury, MA, USA). Creatine was obtained from BLD Pharmatech (Shanghai, China). Methanol was purchased from FUJIFILM Wako Pure Chemical and was distilled before use. Liquid-chromatography-grade acetonitrile was purchased from Kanto Chemical Co., Ltd. (Tokyo, Japan). Ultrapure water was prepared using a Puric-α (Organo Corporation, Tokyo, Japan).

### 2.2. LC/MS/MS Equipment

For G-Met, a hybrid quadrupole time-of-flight tandem mass spectrometer (TripleTOF 5600; SCIEX, Framingham, MA, USA) was connected to an ultra-high-performance liquid chromatograph (Nexera; Shimadzu, Kyoto, Japan) [63]. The measurements were conducted in the positive ion mode using Analyst TF 1.6 software (SCIEX). Peak intensity analysis was conducted using peak processing with MS-DIAL 4.90 software [64]. Multivariate analysis was conducted using MetaboAnalyst version 5.0 [65], and peaks with a *p*-value of less than 0.05 and an at least 2-fold peak intensity ratio between the WT and knockout (KO) samples were extracted using a volcano plot [65]. Analyses were performed according to standard procedures. Statistical analysis was performed and no special treatment, including normalization, was performed.

A QTRAP 6500 quadrupole linear ion-trap hybrid tandem mass spectrometer (SCIEX) with an ESI probe attached to the ion source was used in conjunction with a Nexera series ultra-high-performance liquid chromatograph. The measurements were conducted in positive ion mode. The measurements were conducted using Analyst 1.6.2 (SCIEX). Quantitative analysis was conducted using peak processing in MultiQuant 2.1.1 (SCIEX). The statistical significance testing of peak intensities was conducted using JMP Pro 17.0 (SAS Institute Inc., Cary, NC, USA). In the context of enrichment analysis, candidate metabolic pathways with the potential for metabolic changes were extracted using MetaboAnalyst version 5.0 developed by University of Alberta (Edmonton, AB, Canada) [65].

### 2.3. LC/MS/MS Condition for Global Metabolomics

G-Met LC/high-resolution MS (HRMS) was conducted in positive ion mode. The ion source parameters of G-Met are listed in Table S1. Mobile phase A consisted of formic acid/water (0.1:100, *v*/*v*) and mobile phase B consisted of a mixture of formic acid/acetonitrile/methanol (0.1:40:60, *v*/*v*/*v*). The flow rate was set to 0.4 mL/min in gradient mode, with the ratio altered, as shown in Table S2. InertSustain AQ-C18 (GL Science, 2.1 mm i.d. × 150 mm, 1.9 µm) was utilized as the column, and the column temperature was set to 45 °C. Five microliters of the sample solution was injected.

### 2.4. Cell Culture Conditions

We used disease model cells from the human hepatic cell line HepG2. The cells used in previous studies and in this study were similar [54]. Three types of cells were cultured: two types of HepG2 hepatocellular carcinoma-derived NPC model cells, in which part of *NPC1* (KO1:238-257, 1357-1376 bp, KO2:279-301, 3589-3611) was knocked out using the CRISPR/Cas9 method. The cells were maintained in a culture medium containing DMEM (High Glucose) (Nacalai Tesque) supplemented with 10% fetal bovine serum (Gibco, Thermo Fisher Scientific Inc., Waltham, MA, USA). The cell culture medium consisted of DMEM supplemented with 0.5 mg/mL G418 disulfate (Nacalai Tesque). The cell culture was conducted at 37 °C in a 5% CO_2_ incubator in a 95% air atmosphere.

### 2.5. Global Metabolomics Procedure for Cell Samples and Selection of Metabolites for Targeted Metabolomics

The cultured cells were washed, collected in phosphate-buffered saline (PBS), and sonicated in cold methanol. After the supernatant was dried, it was re-dissolved in methanol to a concentration of 2 million cells/mL for analysis. Samples prepared from the three cell types were combined in equal proportions to prepare a quality control (QC) sample that was then subjected to analysis. At the beginning of the batch analysis, the QC samples were analyzed 20 times, and the measurement samples were analyzed in triplicate in a randomized order: WT, KO1, and KO2. The analytical software Analyst TF 1.6 (SCIEX) was employed for LC/MS analysis and data collection. The data were imported into MS-DIAL 4.90, and the peaks were selected and aligned. The following ions were selected in the positive ion mode: [M + H]^+^, [M + H − H_2_O]^+^, and [M + NH_4_]^+^. Candidate compounds were listed based on the ESI (+)-MS/MS database using authentic standards (16,481 unique compounds) included in MS-DIAL 4.90 [64]. Analyses were performed according to standard procedures. The accurate mass tolerance (MS1) for peak detection, alignment, and identification was set at 0.02 Da, and the retention time tolerance for alignment was set at 0.5 min. To analyze the quantitative changes in G-Met measurements, we used a volcano plot that simultaneously expressed the rate of change in peak intensity and statistical significance. The vertical axis of the volcano plot (−log10 (*p*-value)) indicates the reliability of the peak, and the value on the horizontal axis, log2 (fold change), indicates the intensity ratio of the peak using MetaboAnalyst 5.0 [65]. The peaks located at the upper right of the volcano plot indicate high values in each NPC model cell and the peaks located at the upper left indicate high values in the WT cells. Using this graph, we searched for peaks with intensity differences between KO1, KO2, and the WT. In this study, we considered that metabolites showing significant differences between the two groups were involved in the metabolic changes in NPC and extracted peaks that satisfied *p* < 0.05 and fold change > 2 or <0.5, for KO1 vs. WT and KO2 vs. WT. For the peaks that showed variation between the two, we selected the metabolites to be measured using targeted metabolomics under the condition that the samples could be purchased for identification and quantification. The significance of metabolites or metabolic pathways is well known, and a similar trend was observed for KO1 and KO2 (four metabolites: arginine, tyrosine, glutathione, and creatine).

### 2.6. Standard Solutions for Targeted Metabolomics

Approximately 10 mg of the target compound was dissolved in each solvent and diluted with water/methanol (1:1, *v*/*v*), and the resulting solution was utilized for T-Met analysis. The initial concentrations of the stock solutions and solvents are listed in Table S3. Internal standards (ISs) were diluted in this solution. Dilution was conducted using a 1:1 (*v*/*v*) mixture of water and methanol, with the isotope content being considered, and the solution was diluted to 1 µg/mL. The prepared solutions were stored at 4 °C.

### 2.7. Optimization of LC/MS/MS Conditions for Targeted Metabolomics

To optimize the SRM conditions for both the positive and negative ion modes, 4 μg/mL of each standard solution was injected at a flow rate of 7 μL/min. To determine the MS ion source parameters, the parameters used for the declustering potential (DP) and entrance potential (EP) of the compounds were obtained previously. The final SRM conditions are listed in Table S4.

MS/MS measurements were conducted in the positive ion detection mode. The ion source parameters were optimized using flow injection analysis (the results are presented in Table S5). The mobile phase A, comprising formic acid and water in a 0.1:100 (*v*/*v*) ratio, and the mobile phase B, a mixture of formic acid, acetonitrile, and methanol in a 0.1:50:50 (*v*/*v*/*v*) ratio, were delivered at a flow rate of 0.4 mL/min with an equal ratio of mobile phase A and B. The standard compounds used in this study were glutamine, creatine-^2^H_3_, tryptophan, glutamic acid, methionine, serine, and arginine-[^13^C_6_,^15^N_4_] hydrochloride. CAD was examined at 1 psi intervals from 7 to 11 psi, and CUR at 5 psi intervals from 20 to 55 psi. GS1 and GS2 were examined at 10 psi intervals from 30 to 90 psi. The ISV was examined at 500 V intervals from 4000 to 5500 V, and the TEM was examined at 50 °C intervals from 300 to 700. The ion source parameters used in each operational mode are listed in Table S5. Formic acid: water (0.1:100, *v*/*v*) and formic acid: methanol (0.1:100, *v*/*v*) were used as the mobile phases A and B, respectively. The flow rate was set to 0.3 mL/min, and gradient mode was used to vary the ratio (Table S6). A CAPCELL PAK ADME HR column (2.1 × 50 mm, 2 µm) was used, and the column temperature was set at 45 °C. A 1 µL sample solution was injected. Optimized ion source parameters (Table S5), SRM transitions (Table S4), and LC gradient conditions (Table S6) were applied to the targeted metabolomics method.

### 2.8. Calibration Curves for Targeted Metabolomics

A standard solution was prepared by mixing and diluting the components to obtain standard solutions for the calibration curves of the standard mixed solution. Each compound was then diluted to achieve concentrations of 1, 2.5, 5, 7.5, 10, 25, 50, 75, 100, 250, 500, 750, 1000, 2500, 5000, 7500, 12,500, 15,000, 20,000, 25,000, 50,000, 75,000, and 100,000 ng/mL, respectively. Calibration curves were prepared by mixing equal volumes of the standard and IS solutions in a vial. Subsequently, 1 µL of the resulting mixture was injected into the LC-MS/MS system. The peak area ratios of the analyte and IS at each concentration were plotted and a calibration curve was prepared using the least-squares method with 1/x weighting.

### 2.9. Cell Sample Preparation for Targeted Metabolomics

The cultured cells were washed and collected in PBS. The collected cells were sonicated in a solution of water and methanol (1:1 *v*/*v*). Following supernatant removal, the cells were reconstituted in a solution of water and methanol (1:1, *v*/*v*) at a concentration of 10,000 cells per 1 µL. Equal volumes of the aforementioned solution and the IS solution were combined and transferred to a vial. Subsequently, 1 µL was injected for analysis in positive ion mode.

### 2.10. Quantification of Metabolites by Targeted Metabolomics

Peak integration was conducted using MultiQuant 2.1.1 (SCIEX), and data were collected using Analyst software version 1.6.2.

The sample concentrations were determined using a calibration curve. A Student’s *t*-test was conducted in JMP Pro 17.0 (SAS Institute, Cary, NC, USA) to ascertain whether there were significant differences in the concentration of metabolites.

### 2.11. Variation Analysis of Metabolic Pathways

Metabolic pathway analysis was conducted using the compound concentrations obtained from quantitative analysis. Enrichment analysis was conducted using MetaboAnalyst 5.0, eliminating the distinction between enantiomers of the compound names. Common metabolic pathways were extracted from KEGG metabolic pathways by comparing the WT, KO1, and KO2 mice. Student’s *t*-test was performed using JMP Pro, and enrichment analysis was performed using MetaboAnalyst for metabolites with *p*-values < 0.05, without special treatment, including normalization.

## 3. Results and Discussion

### 3.1. Global Metabolome Analysis of NPC Model Cells

First, cell samples were subjected to G-Met analysis. Because NPC exhibits symptoms in the liver, the HepG2 cell line, which is the most popular human-derived hepatic cell line, was used in this study. As NPC model cells, hepG2 cell lines with *NPC1* knocking out based on CRISPR/Cas9 gene editing were used. Two types of NPC models, KO1 and KO2, have been previously developed [54]. These have different knockout sites. The NPC model cell line KO1 has exon skipping at positions 238–257 (exon 1) and 1357–1376 bp (exon 8, transmembrane region, and extracellular loop). In contrast, the NPC model cell line KO2 has exon skipping at 279–301 bp (exon 1) and 3589–3611 bp (exon 22, transmembrane loop region) [54]. As reported in our previous study, both common and distinct proteomic alterations were observed [54]. Therefore, we performed a metabolomic analysis of the two NPC cell lines.

**Figure 2:**
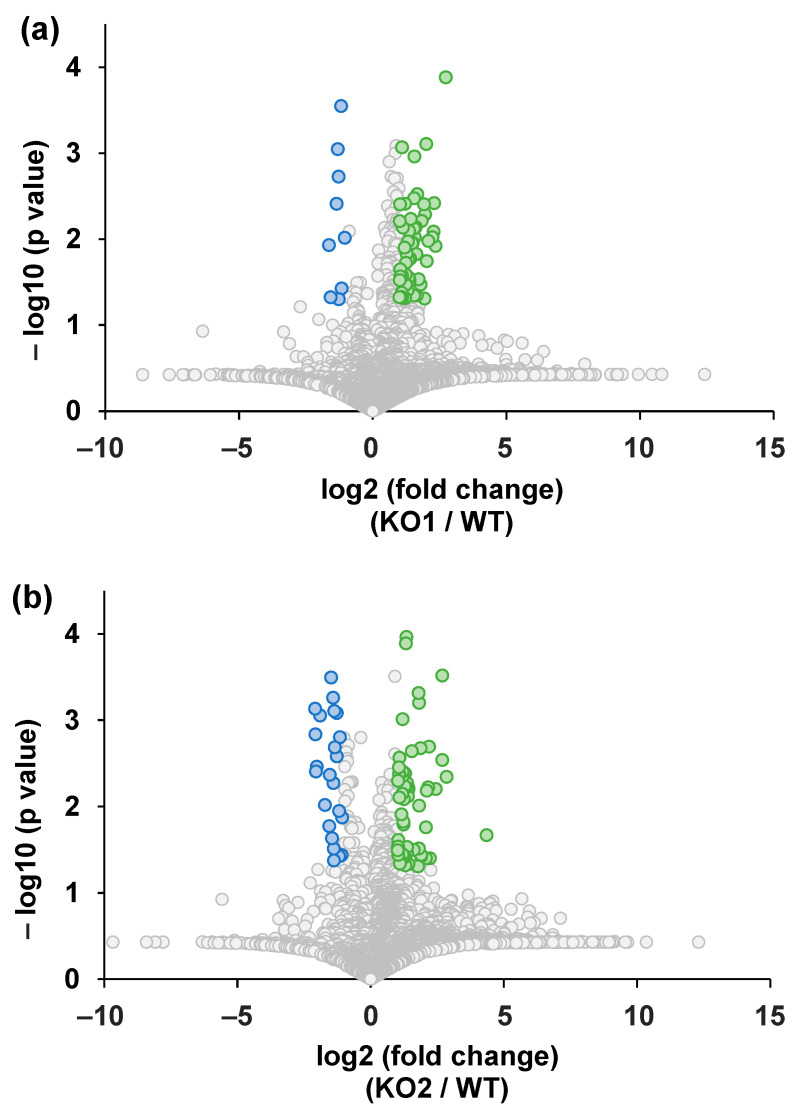
Volcano plots from the results of global metabolomics analysis: (**a**) KO1 vs. WT; (**b**) KO2 vs. WT. Green-colored plots—greater than 2-fold increased and *p* < 0.05 metabolites in NPC model cells; blue-colored plots—greater than 0.5-fold decreased and *p* < 0.05 metabolites in NPC model cells; gray-colored plots—non-differentially changed metabolites.

**Figure 3:**
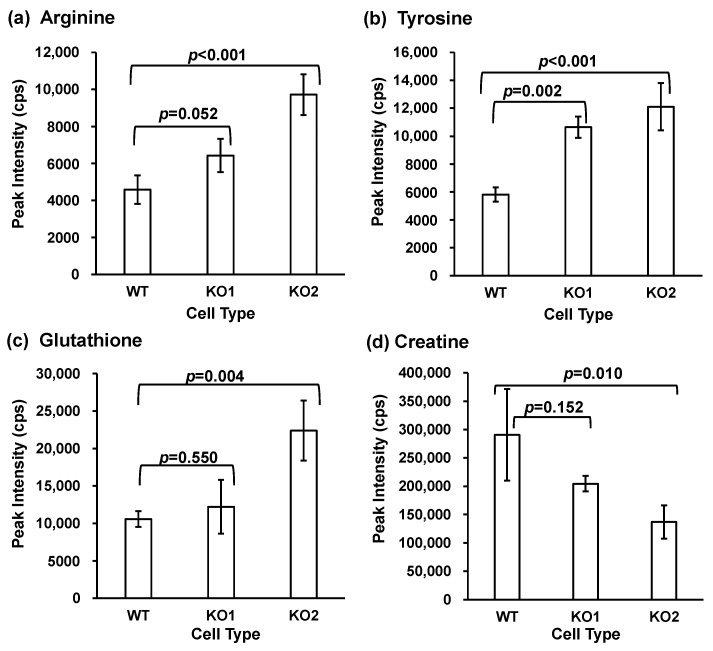
Peak intensities of cellular metabolites using global metabolomics. (**a**) Arginine; (**b**) tyrosine; (**c**) glutathione; (**d**) creatine. Bars represent the mean and error bars represent standard deviations. *p*-values between the WT and NPC model cell lines are shown for each item.

Random MS signal alterations are generally observed in G-Met analysis [66,67]. Therefore, the MS signal correction is performed using various methods [68,69,70]. In this study, we adopted intermittent QC analysis in batches to correct for signal alterations [71]. After 20 QC samples were measured, cell samples (WT, KO1, and KO2) were measured in a random order [63,71]. The LC-MS data obtained were imported into two software packages. One was MS-DIAL version 4.9 for data integration [64] and the other was MetaboAnalyst for statistical analyses in the alteration analysis [65,72,73]. A volcano plot was used to analyze quantitative alterations in G-Met analysis. All detected peaks are summarized (Table S7), and the number of peaks extracted for each peak is presented in Figure S1. In this study, we found that metabolites exhibiting significant differences between the two groups were involved in the metabolic alterations in NPC. The peaks located in the upper right of the volcano plot indicate elevated amounts in each NPC model cell line (KO1 or KO2), whereas the peaks located in the upper left indicate elevated amounts in the WT cells (Figure 2). Subsequently, KEGG databases were used to identify the list of altered peaks. Using the following criteria: the availability of reference materials for identification and quantification, the documented significance of the metabolite or metabolic pathway, and the observation of a similar trend in KO1 and KO2, we focused on four metabolites (arginine, tyrosine, glutathione, and creatine). Figure 3 shows the peak intensities of the four metabolites, and the results indicate alteration trends in the NPC model cell lines compared to the WT cell lines. The peak intensities of arginine, tyrosine, and glutathione increased in both KO cell lines relative to WT, whereas creatinine decreased in the NPC model cell lines. Next, to confirm the quantitative alteration of these changes in the G-Met results, we quantified the metabolites produced by T-Met using LC-SRM/MS analysis.

### 3.2. Targeted Metabolomics for Quantification

Subsequently, T-Met in NPC model cells was analyzed using LC-MS/MS. We selected 4 metabolites (arginine, tyrosine, glutathione, and creatine) and 12 related metabolites (carnitine, creatinine, cysteine, cysteine, glutamic acid, glycocyamine, methionine, ornithine, proline, serine, and tryptophan) as analytes (Table 1).

**Figure 4:**
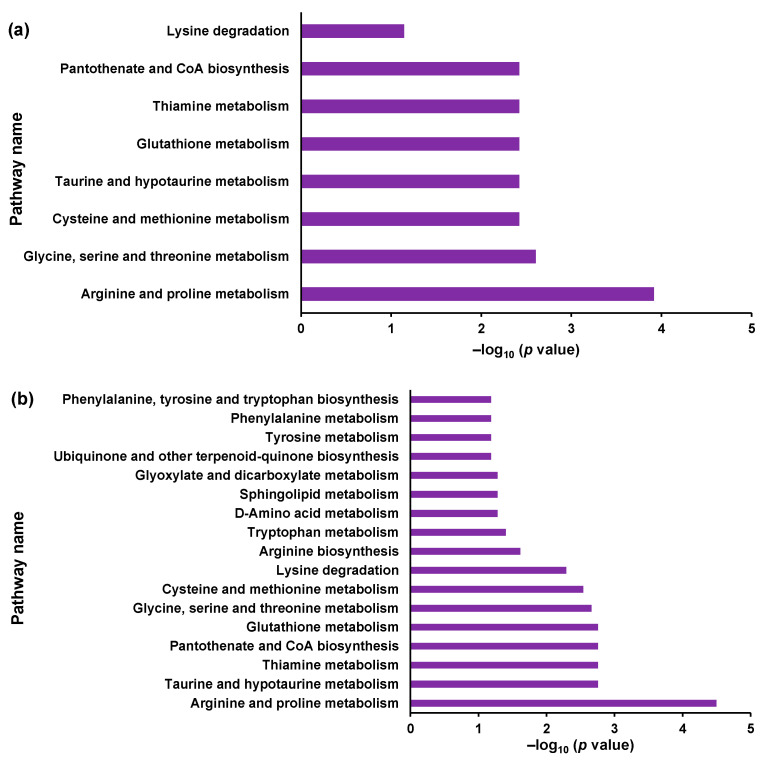
Pathways selected from enrichment analysis by compounds meeting *p* < 0.05 between groups: (**a**) WT vs. KO1; (**b**) WT vs. KO2. Metabolic pathways to which molecules with significant changes are mapped are extracted from the KEGG database using MetaboAnalyst.

First, the MS/MS conditions for the analytes and ISs were optimized (Table S4). Subsequently, the ionization parameters were optimized by flow injection analysis (FIA) (Table S5). The calibration curves exhibited a wide linearity range (Table 1). The concentrations of each metabolite in the WT, KO1, and KO2 cell samples were determined. The metabolite concentrations are listed in Table 2. All metabolites were quantified except for glycocyamine, which is an intermediate between arginine and creatinine. Four and ten metabolites were significantly different between KO1 and WT cell lines and KO2 and WT cells, respectively (Table 2). Subsequently, altered metabolic pathways were investigated by enrichment analysis using MetaboAnalyst 5.0 [65]. The significantly altered KEGG metabolic pathways are shown in Figure 4 [74,75]. The extracted metabolic pathways are presented in Figure 4 and Table S8. Among the pathways with common alterations in KO1 and KO2, we focused on three pathways: arginine and proline metabolism (map00330), glycine, serine, and threonine metabolism (map00260), and cysteine and methionine metabolism (map00270).

**Figure 5:**
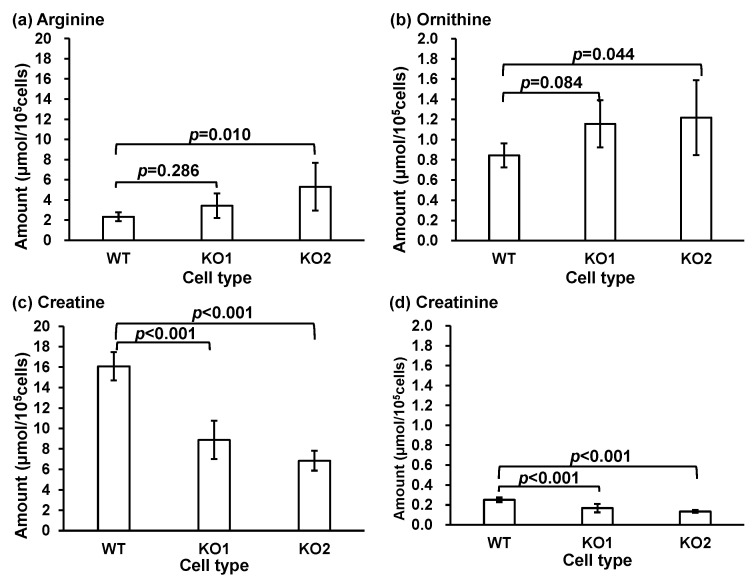
The amount of metabolites related to the arginine and proline metabolism pathways in cells: (**a**) arginine, (**b**) ornithine, (**c**) creatine, and (**d**) creatinine. Bars represent the mean and error bars represent standard deviations.

Arginine and proline metabolism was the most significant pathway in both KO1 and KO2 (Figure 4). The arginine, ornithine, creatine, and creatinine levels were quantified. The pathways involved in arginine and proline metabolism (map00330) are shown in Figure S3. The quantified T-Met results for arginine and proline metabolisms are shown in Figure 5. Arginine, situated upstream of the metabolic pathway, increased in the model cells, whereas creatine and creatinine, located downstream, decreased. A reduction in creatinine levels was observed in a liver damage model [76]. Anomalies in the arginine, ornithine, creatine, and creatinine metabolic pathways may occur during liver damage. These pathways have been associated with amyotrophic lateral sclerosis [77], asthma [78,79], and cysteinosis [80,81]. These diseases are associated with various inflammatory conditions. NPC patients exhibit signs of liver inflammation [82]. These metabolites may be associated with pathological and inflammatory conditions. In contrast, elevated arginine and ornithine levels suggest the disruption of the urea cycle, which could lead to the accumulation of toxic ammonia and contribute to disease progression and patient prognosis. Increased arginine and ornithine levels can also increase NO production, which may exacerbate oxidative stress and inflammation and potentially worsen the disease [83]. Decreased creatinine levels may suggest impaired energy metabolism and mitochondrial dysfunction, both of which are critical for neurodegenerative processes [84,85]. Our previous report also suggested that the decline in steroid hormone levels results from mitochondrial dysfunction [86].

**Figure 6:**
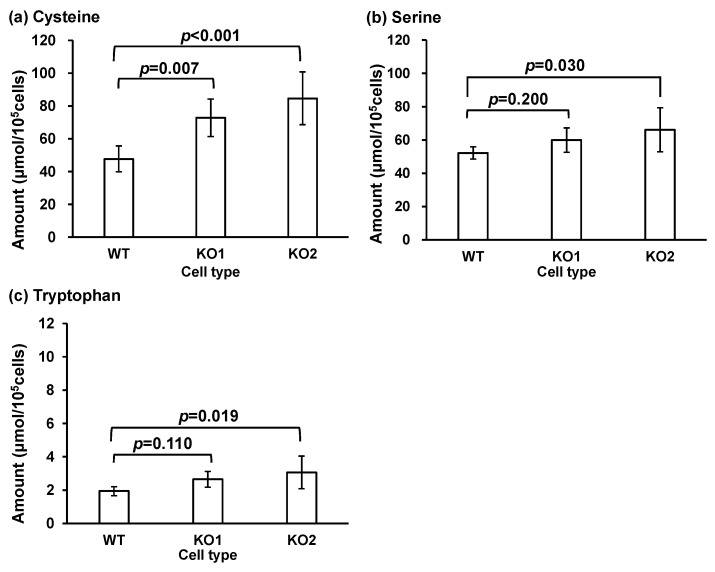
The amounts of metabolites related to the glycine, serine, and threonine metabolism pathways in cells: (**a**) cysteine, (**b**) serine, and (**c**) tryptophan. Bars represent mean and error bars represent standard deviations.

**Figure 7:**
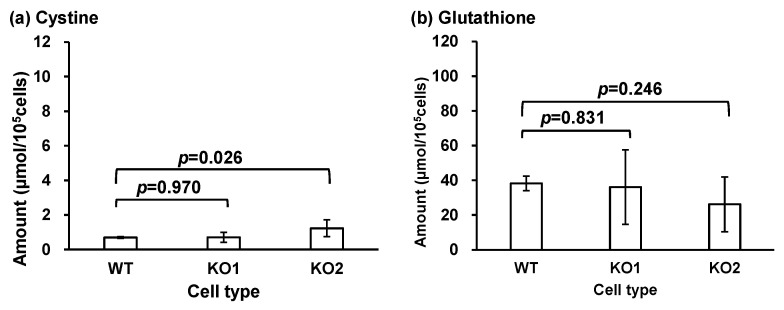
The amounts of metabolites related to cysteine and methionine metabolism pathways in cells: (**a**) cystine and (**b**) glutathione. Bars represent the mean, and error bars represent standard deviations.

Next, the glycine, serine, and threonine metabolism pathways (map00260), as well as serine, tryptophan, cysteine, and creatine, were considered. The glycine, serine, and threonine metabolic pathways (map00260) are shown in Figure S4. The quantitative values of the metabolites are shown in Figure 6. Changes were observed in KO1 and KO2 for serine and cysteine and in WT and KO2 for tryptophan and serine. Increased cysteine levels may indicate an imbalance in redox homeostasis because cysteine is a precursor of the antioxidant glutathione [87,88]. In our study, glutathione levels did not change (Figure 7b). This may indicate a compensatory response to increased oxidative stress in the NPC. However, if oxidative stress overwhelms antioxidant defenses, it can accelerate cellular damage and worsen prognosis. The relationship between oxidative stress and oxysterols has been reported [38,39,89]. A surplus of reactive oxygen species exceeding scavenging capacity results in the accumulation of oxysterols. Elevated serine levels may indicate alterations in one-carbon metabolism, which is important for DNA synthesis, repair, and methylation. Increased serine levels also suggest a disruption in neurotransmitter synthesis, particularly in the production of glycine and serine, which are involved in neurotransmission and neuroprotection [90,91,92]. These changes may reflect compensatory mechanisms or pathological changes that influence disease course. Elevated tryptophan levels may disrupt metabolic pathways such as the kynurenine pathway, which is involved in neuroinflammation and neurotoxicity. Increased tryptophan levels may also be linked to alterations in serotonin synthesis, thereby affecting mood and cognitive function in patients with NPC [93]. These changes may be correlated with neuropsychiatric symptoms and disease progression. L-Serine is a raw material used for ceramide and sphingomyelin synthesis [52,94,95,96]. Sphingomyelin and lysosphingomyelin levels are increased in NPC [44,97,98,99]. Moreover, the levels of PPCS, a serine derivative, are increased in NPC [37,46,48,49,50,51]. Therefore, increased serine levels may be involved in NPC pathogenesis.

Next, we considered cysteine and methionine metabolism, and serine, cystine, cysteine, and glutathione metabolism (map00270, Figure S5). The quantitative results of the metabolites in the map are shown in Figure 7 (although cysteine has already been shown in Figure 6a). Alterations in cysteine, cysteine, and glutathione levels were observed in both KO1 and KO2 types. This pathway is involved in the synthesis of sulfur-containing molecules that play an important role in the regulation of antioxidant molecules, such as glutathione, and in physiological changes in the inflammatory process in various organs, including the nervous system [88,100]. The cystine/glutamate antiporter pathway is involved in neuronal degeneration [101]. An increase in oxidative stress and the resulting increase in oxysterols and metabolites has been reported in NPC [38,39,40,89,102,103,104,105,106,107,108,109]. In this study, changes in glutathione levels were not significant in the G-Met group; therefore, they were not quantified. However, this change in pathway metabolites may result from an adaptation to oxidative stress. Further analyses are necessary to elucidate the relationships between NPC, lysosomal abnormalities, and these metabolites.

## 4. Conclusions

In this study, we performed a metabolomic analysis of NPC cell lines to elucidate the pathological molecular mechanisms of NPC. First, we subjected NPC cell line samples to G-Met analysis and discovered alterations in some metabolites. We focused on arginine, tyrosine, glutathione, and creatine, and performed T-Met analysis on four metabolites and assessed twelve related metabolites by quantitative analysis. T-Met analysis indicated that metabolome analysis using LC-MS/MS has the potential to elucidate the various molecular mechanisms underlying NPC. The findings of this study will facilitate the elucidation of new pathological molecular mechanisms and advance drug discovery research.

## Figures and Tables

**Table 1:** Calibration curves for targeted metabolomics.

Analyte	Equation	Correlation Coefficient (R^2^)	Range (ng/mL)	IS
Arginine	y = 0.75221x + 0.00341	0.9987	50–5000	Arginine-[^13^C_6_,^15^N_4_]
Carnitine	y = 1.18022x + 0.04072	0.9945	75–2500	Arginine-[^13^C_6_,^15^N_4_]
Creatine	y = 0.38279x − 0.00191	0.9993	50–7500	Creatine-[^2^H_3_]
Creatinine	y = 0.68207x + 0.00105	0.9962	5–250	Arginine-[^13^C_6_,^15^N_4_]
Cysteine	y = 0.13442x − 0.16923	0.9959	2500–25,000	Arginine-[^13^C_6_,^15^N_4_]
Cystine	y = 0.08118x − 0.00016050	0.9947	10–2500	Creatine-[^2^H_3_]
Glutamic acid	y = 0.18196x − 0.42880	0.9940	5000–50,000	Creatine-[^2^H_3_]
Glutamine	y = 0.29085x − 0.03538	0.9975	500–12,500	Creatine-[^2^H_3_]
Glutathione	y = 0.22382x − 0.36368	0.9869	2500–20,000	Arginine-[^13^C_6_,^15^N_4_]
Glycocyamine	y = 0.07604x − 0.00212	0.9989	100–75,000	Creatine-[^2^H_3_]
Methionine	y = 0.10443x − 0.00765	0.9937	100–15,000	Creatine-[^2^H_3_]
Ornithine	y = 0.38240x + 0.00333	0.9943	10–1000	Arginine-[^13^C_6_,^15^N_4_]
Proline	y = 0.86494x − 0.00627	0.9970	250–10,000	Creatine-[^2^H_3_]
Serine	y = 0.06414x − 0.05882	0.9963	2500–25,000	Arginine-[^13^C_6_,^15^N_4_]
Tryptophan	y = 0.36811x − 0.00050155	0.9994	10–1000	Creatine-[^2^H_3_]
Tyrosine	y = 0.58450x − 0.16496	0.9972	750–20,000	Creatine-[^2^H_3_]

The calibration curve range was set to include the sample concentrations to be quantified in subsequent experiments. All the equations were fitted to a linear model.

**Table 2:** The amounts of metabolites in cells.

Compound	WT Cells (µmol/10^5^ Cells)	KO1 Cells (µmol/10^5^ Cells)	KO2 Cells (µmol/10^5^ Cells)
Arginine	2.33 ± 0.43	3.43 ± 1.22	5.32 ± 2.37 *
Carnitine	1.29 ± 0.19	0.95 ± 0.31 *	0.95 ± 0.04 *
Creatine	16.1 ± 1.38	8.88 ± 1.87 ***	6.84 ± 0.97 ***
Creatinine	0.25 ± 0.02	0.17 ± 0.04 ***	0.13 ± 0.01 ***
Cysteine	47.7 ± 7.89	72.8 ± 11.5 **	84.7 ± 16.1 ***
Cystine	0.70 ± 0.05	0.71 ± 0.29	1.23 ± 0.49 *
Glutamic acid	99.4 ± 13.2	89.5 ± 17.7	95.1 ± 5.51
Glutamine	7.28 ± 1.01	8.25 ± 1.92	10.5 ± 3.76
Glutathione	38.2 ± 4.15	36.1 ± 21.4	26.2 ± 15.8
Glycocyamine	N.D.	N.D.	N.D.
Methionine	6.63 ± 0.94	7.56 ± 1.72	8.79 ± 2.95
Ornithine	0.84 ± 0.12	1.16 ± 0.23	1.22 ± 0.37 *
Proline	43.3 ± 4.55	36.7 ± 5.55	38.7 ± 5.72
Serine	52.2 ± 3.71	59.9 ± 7.35	66.1 ± 13.2 *
Tryptophan	1.94 ± 0.27	2.65 ± 0.48	3.06 ± 0.98 *
Tyrosine	8.34 ± 1.09	10.3 ± 1.92	11.8 ± 3.45 *

Mean and standard deviation of the concentration of each metabolite were calculated. N.D., not detected. * means *p* < 0.05, ** means *p* < 0.01, *** means *p* < 0.001.

## Data Availability

Data are presented in the article or supplementary material.

## Supplementary Materials

The following information was downloaded from https://www.mdpi.com/article/10.3390/metabo14100515/s1, Figure S1: Altered metabolites in NPC model cell lines; Figure S2: Total number of peaks detected and number of peaks with variation in non-targeted metabolomics in NPC model and wild-type (WT) cells; Figure S3: Metabolic pathway analysis of arginine and proline metabolism; Figure S4: Metabolic pathway analysis of glycine, serine, and threonine metabolism; Figure S5: Metabolic pathway analysis of cysteine and methionine metabolism; Table S1: Ion source parameters for global metabolomics of cells. Table S2: Gradient programs for non-targeted metabolomics of cells. Table S3: Stock solutions of standard T-Met solutions. Table S4: Optimized SRM parameters for targeted metabolomics. Table S5: Optimized ionization parameters for targeted metabolomics; Table S6: Gradient programs for targeted metabolomics of cells; Table S7: Peak list of global metabolomics for cells; Table S8: Pathways selected from the enrichment analysis of compounds that showed significant differences.
