# Supplementary material for: Global and Targeted Metabolomics for Revealing Metabolomic Alteration in Niemann-Pick Disease Type C Model Cells
Source: Metabolites. 2024 Sep 24;14(10):515. doi: 10.3390/metabo14100515 (PMC11509386; doi:10.3390/metabo14100515)
Supplement: Supplementary file 1 [file metabolites-14-00515-s001.zip › Figures S1-S5.pptx]

## Slide 1
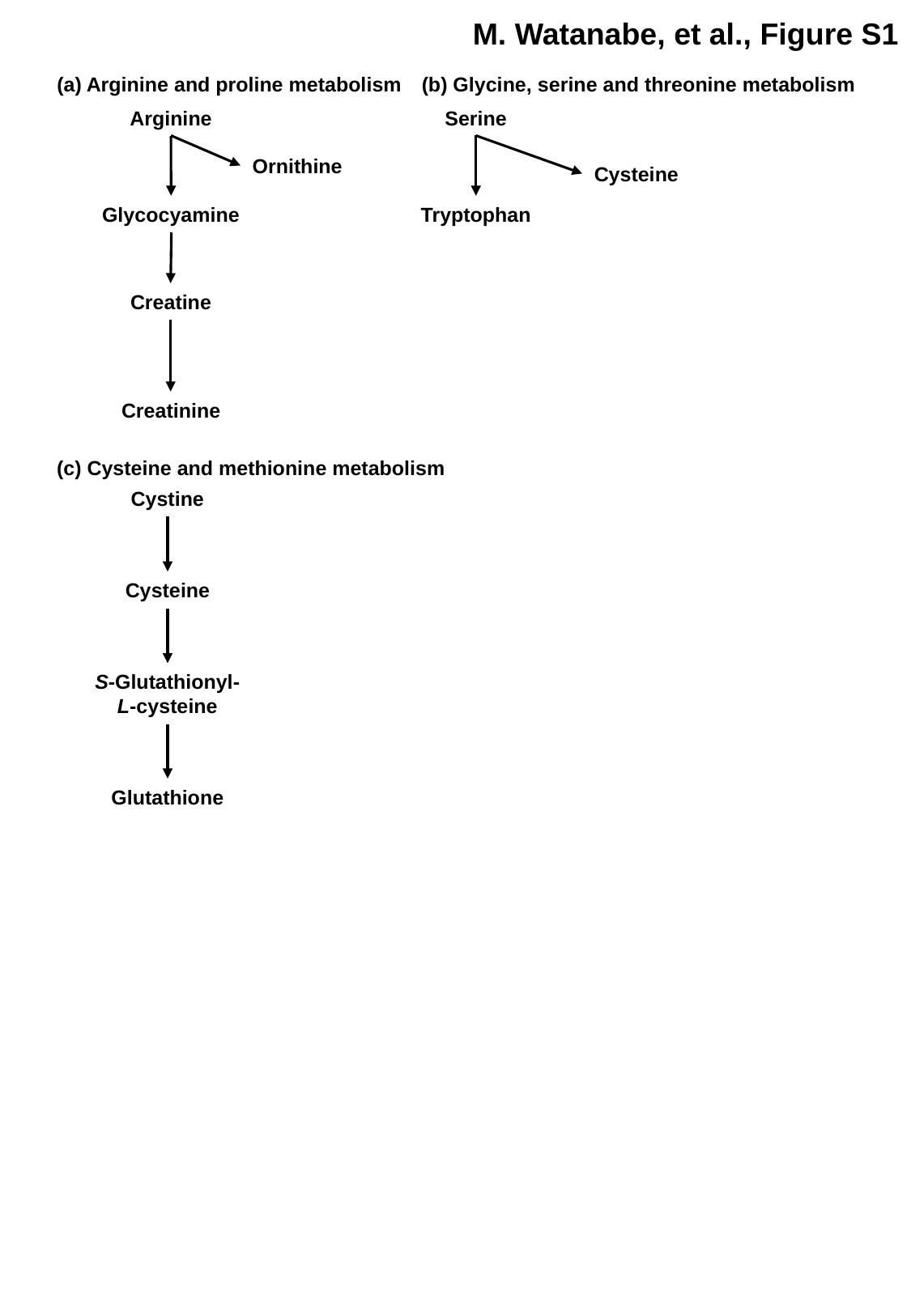

# M. Watanabe, et al., Figure S1
(b) Glycine, serine and threonine metabolism
(a) Arginine and proline metabolism
Arginine
Serine
Ornithine
Cysteine
Glycocyamine
Tryptophan
Creatine
Creatinine
(c) Cysteine and methionine metabolism
Cystine
Cysteine
S-Glutathionyl-
L-cysteine
Glutathione

## Slide 2
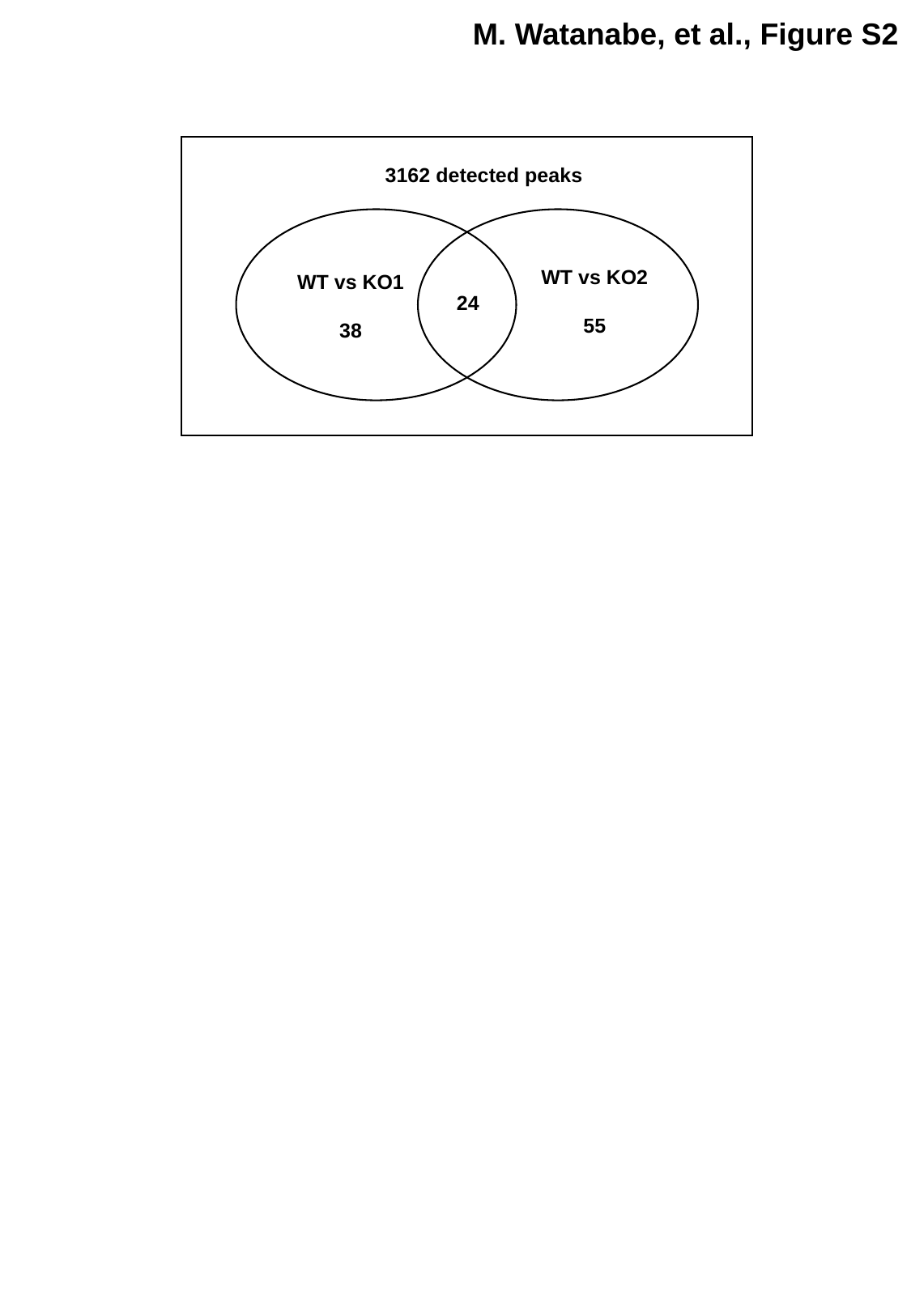

# M. Watanabe, et al., Figure S2
3162 detected peaks
WT vs KO2
55
WT vs KO1
38
24

## Slide 3
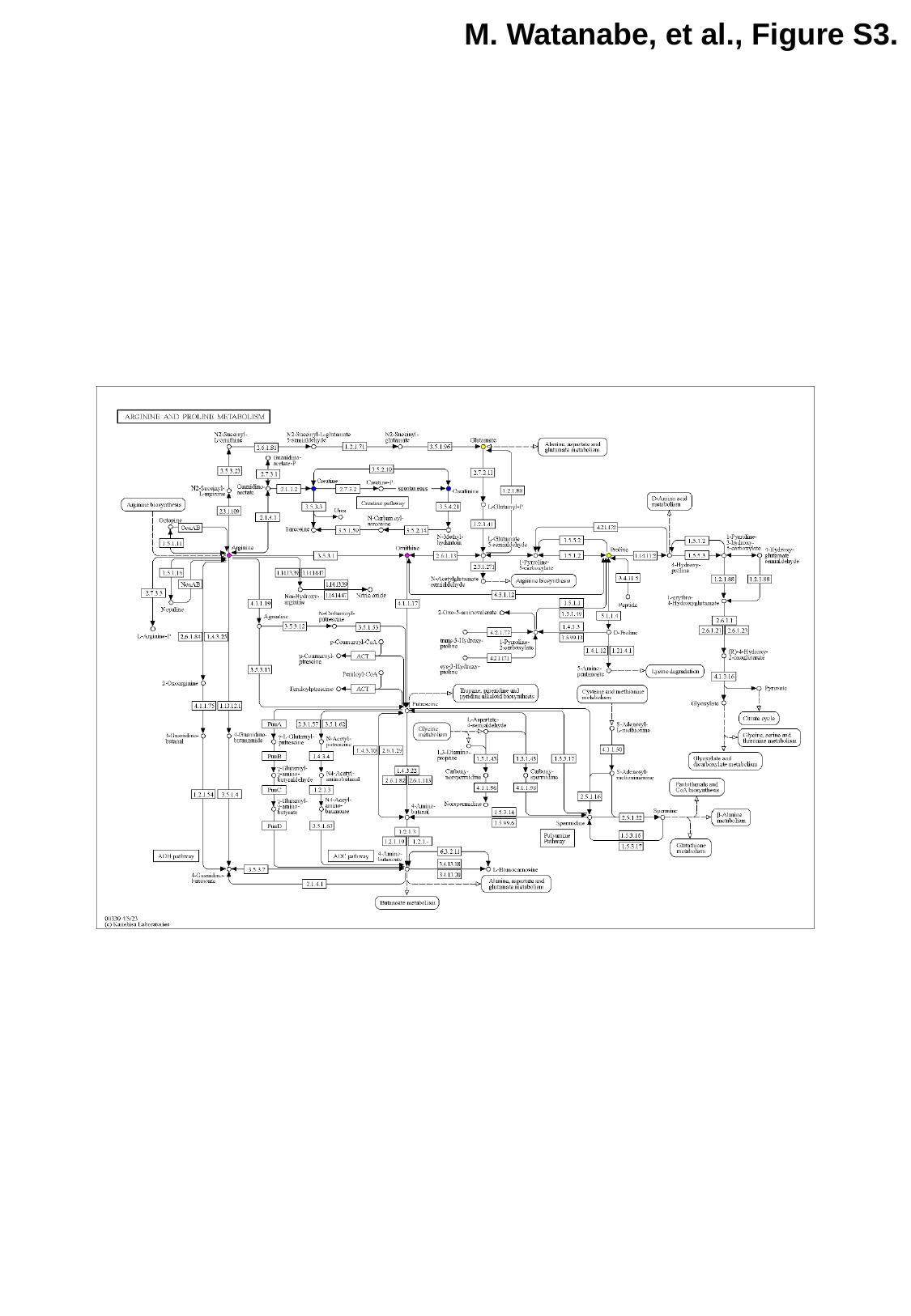

# M. Watanabe, et al., Figure S3.

## Slide 4
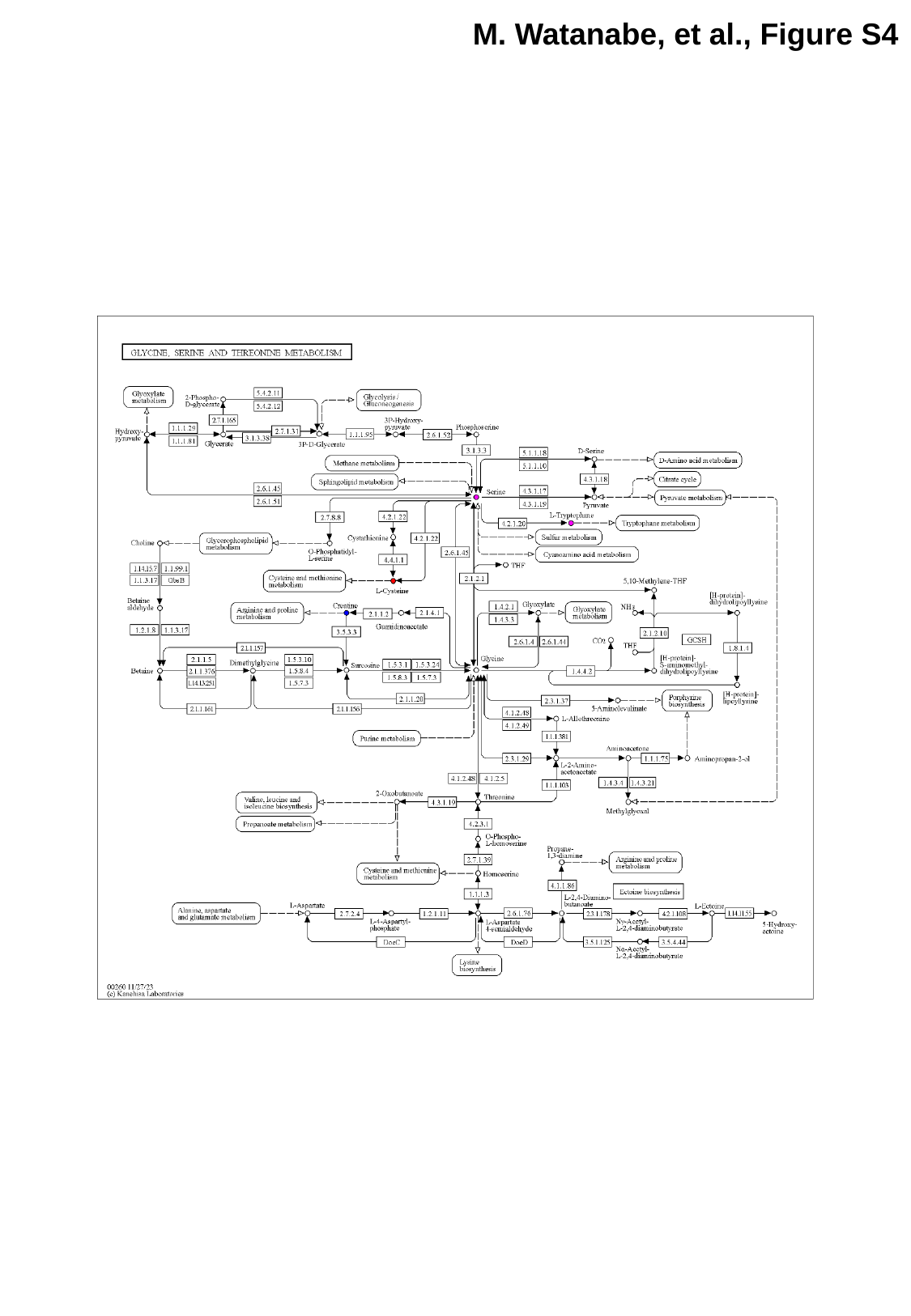

# M. Watanabe, et al., Figure S4

## Slide 5
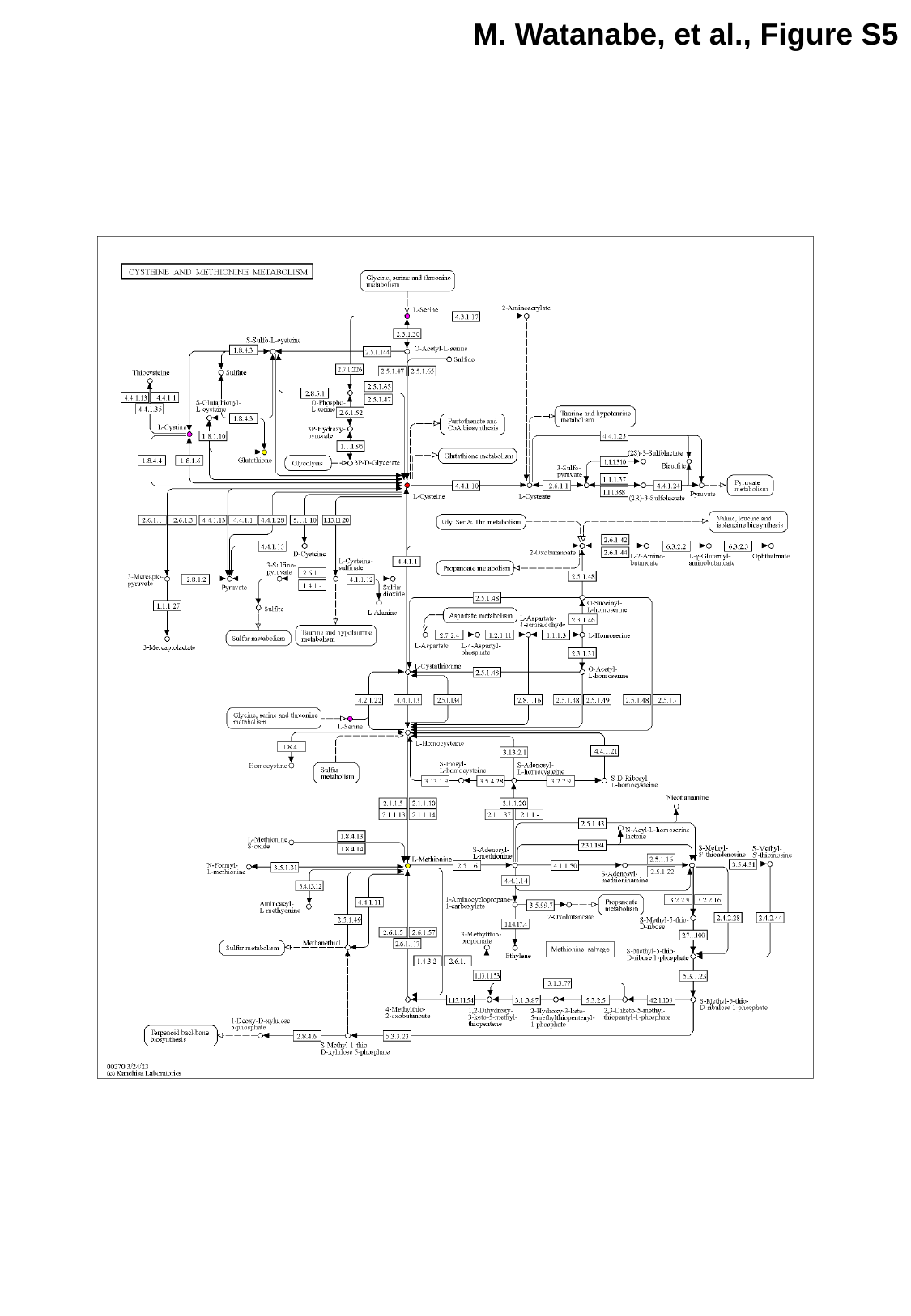

# M. Watanabe, et al., Figure S5
