# Supplementary material for: Global and Targeted Metabolomics for Revealing Metabolomic Alteration in Niemann-Pick Disease Type C Model Cells
Source: Metabolites. 2024 Sep 24;14(10):515. doi: 10.3390/metabo14100515 (PMC11509386; doi:10.3390/metabo14100515)
Supplement: Supplementary file 1 [file metabolites-14-00515-s001.zip › Table S1.pdf]

Table S1. Ion source parameters for global metabolomics of cells.

Item (unit)	Value
IonSpray voltage floating (V)	5500
Declustering potential (V)	100
Collision energy (V)	10
Ion source gas 1 (psi)	40
Ion source gas 2 (psi)	40
Temperature (°C)	550
MS range (*m/z*)	70 to 1200
Curtain gas (psi)	35
MS1 accumulation time (msec)	150
MS2 accumulation time (msec)	50
Maximum number of candidate ions to monitor per cycle	8
Cycle time (s)	0.6
