# Supplementary material for: Global and Targeted Metabolomics for Revealing Metabolomic Alteration in Niemann-Pick Disease Type C Model Cells
Source: Metabolites. 2024 Sep 24;14(10):515. doi: 10.3390/metabo14100515 (PMC11509386; doi:10.3390/metabo14100515)
Supplement: Supplementary file 1 [file metabolites-14-00515-s001.zip › Table S3.pdf]

Table S3. Stock solutions of standard T-Met solutions.

Analyte	Concentration (mg/mL)	Solvent
Arginine	1.00	Water/methanol (1:1, v/v)
Carnitine	1.00	Water/28% NH_3 in water (50:1, v/v)
Creatine	1.00	Water
Creatinine	1.00	Water/methanol (1:1, v/v)
Cysteine	1.00	Water/28% NH_3 in water (50:1, v/v)
Cystine	1.02	Water/36% HCl in water (100:1, v/v)
Glutamic acid	1.01	Water
Glutamine	1.01	Water
Glutathione	1.00	Water/28% NH_3 in water (50:1, v/v)
Methionine	1.01	Water
Ornithine	1.00	Water/methanol (1:1, v/v)
Proline	1.02	Water/methanol (1:1, v/v)
Serine	1.00	Water/methanol (1:1, v/v)
Tryptophan	1.00	Water
Tyrosine	1.00	Water/28% NH_3 in water (50:1, v/v)
Arginine-[$^{13}C_6$, $^{15}N_4$]	0.500	Water/methanol (1:1, v/v)
Creatine-[$^{2}H_3$]	1.00	Water
