# Supplementary material for: Global and Targeted Metabolomics for Revealing Metabolomic Alteration in Niemann-Pick Disease Type C Model Cells
Source: Metabolites. 2024 Sep 24;14(10):515. doi: 10.3390/metabo14100515 (PMC11509386; doi:10.3390/metabo14100515)
Supplement: Supplementary file 1 [file metabolites-14-00515-s001.zip › Table S4.pdf]

Table S4. Optimized SRM parameters for targeted metabolomics.

Analyte	Q1 (*m/z*)	Q3 (*m/z*)	DP (V)	EP (V)	CE (V)	CXP (V)
Arginine	175	70	40	12	23	12
Carnitine	162	103	1	10	21	12
Creatine	132	90	40	14	17	4
Creatinine	114	43.9	56	10	21	20
Cysteine	122	76	1	10	17	12
Cystine	241	152	46	10	17	10
Glutamic acid	148	83.8	100	12	23	10
Glutamine	147	130	40	14	13	18
Glutathione	308	179	31	10	17	10
Glycocyamine	118	76	1	10	15	10
Methionine	150	133	100	12	13	8
Ornithine	133	69.9	31	10	21	6
Serine	106	60	100	12	13	10
Tryptophan	205	188	100	12	13	10
Tyrosine	182	165	26	10	13	10
Arginine-[$^{13}C_6$,$^{15}N_4$]	185	75.2	40	12	27	6
Creatine-[$^{2}H_3$]	135	93.1	40	14	17	6
