# Supplementary material for: Global and Targeted Metabolomics for Revealing Metabolomic Alteration in Niemann-Pick Disease Type C Model Cells
Source: Metabolites. 2024 Sep 24;14(10):515. doi: 10.3390/metabo14100515 (PMC11509386; doi:10.3390/metabo14100515)
Supplement: Supplementary file 1 [file metabolites-14-00515-s001.zip › Table S5.pdf]

Table S5. Optimized ionization parameters for targeted metabolomics.

	CUR (psi)	CAD (psi)	IS (V)	TEM (°C)	GS1 (psi)	GS2 (psi)
Positive ion mode	35	11	4000	300	30	30
