# Supplementary material for: Global and Targeted Metabolomics for Revealing Metabolomic Alteration in Niemann-Pick Disease Type C Model Cells
Source: Metabolites. 2024 Sep 24;14(10):515. doi: 10.3390/metabo14100515 (PMC11509386; doi:10.3390/metabo14100515)
Supplement: Supplementary file 1 [file metabolites-14-00515-s001.zip › Table S6.pdf]

Table S6. Gradient programs for targeted metabolomics of cells.

Time (min)	A (%)	B (%)
0	80	20
2	80	20
2.01	0	100
4	0	100
