# Supplementary material for: Global and Targeted Metabolomics for Revealing Metabolomic Alteration in Niemann-Pick Disease Type C Model Cells
Source: Metabolites. 2024 Sep 24;14(10):515. doi: 10.3390/metabo14100515 (PMC11509386; doi:10.3390/metabo14100515)
Supplement: Supplementary file 1 [file metabolites-14-00515-s001.zip › Table S8.pdf]

Table S8. Pathways selected from the enrichment analysis of compounds that showed significant differences.

Pathway	KEGG ID	WT vs KO1	WT vs KO2
Arginine and proline metabolism	map00330	Selected	Selected
Taurine and hypotaurine metabolism	map00430	Selected	Selected
Thiamine metabolism	map00730	Selected	Selected
Pantothenate and CoA biosynthesis	map00770	Selected	Selected
Glutathione metabolism	map00480	Selected	Selected
Glycine, serine and threonine metabolism	map00260	Selected	Selected
Cysteine and methionine metabolism	map00270	Selected	Selected
Lysine degradation	map00310	Selected	Selected
Arginine biosynthesis	map00220		Selected
Tryptophan metabolism	map00380		Selected
Sphingolipid metabolism	map00600		Selected
Glyoxylate and dicarboxylate metabolism	map00630		Selected
Ubiquinone and other terpenoid-quinone biosynthesis	map00130		Selected
Tyrosine metabolism	map00350		Selected
Phenylalanine metabolism	map00360		Selected
Phenylalanine, tyrosine and tryptophan biosynthesis	map00400		Selected

The commonalities of the results are tabulated in Fig. 3. Nine pathways were selected in common.
